# Effective Reproduction Number of Smear-Positive Pulmonary Tuberculosis in Iran: A Registry-Based Study (2011-2021)

**DOI:** 10.34172/jrhs.2024.168

**Published:** 2024-07-30

**Authors:** Maryam Rastegar, Vahid Fakoor, Eisa Nazar, Mahshid Nasehi, Saeed Sharafi, Mohammad Taghi Shakeri

**Affiliations:** ^1^Department of Biostatistics, School of Health, Mashhad University of Medical Sciences, Mashhad, Iran; ^2^Student Research Committee, School of Health, Mashhad University of Medical Sciences, Mashhad, Iran; ^3^Department of Statistics, Ferdowsi University of Mashhad, Mashhad, Iran; ^4^Psychiatry and Behavioral Sciences Research Center, Addiction Institute, Mazandaran University of Medical Sciences, Sari, Iran; ^5^Department of Epidemiology, Iran University of Medical Sciences, Tehran, Iran; ^6^Center for Communicable Diseases Control, Ministry of Health and Medical Education, Tehran, Iran; ^7^Social Determinant of Health Research Center, Mashhad University of Medical Sciences, Mashhad, Iran

**Keywords:** Effective reproduction number, Tuberculosis, Incidence rate, Iran

## Abstract

**Background:** Tuberculosis (TB) remains a major public health issue in Iran, especially smear-positive pulmonary tuberculosis (SPPTB), due to its high transmission rate. Examining the effective reproduction number(*R_t_*) of SPPTB and patient characteristics is crucial for crafting targeted TB control measures. This study aimed to assess the *R_t_* of SPPTB in Iran from 2011 to 2021 and profile SPPTB patient demographics, initial smear bacilli density, diagnosis delays, and spatial distribution.

**Study Design:** This is a historical cohort study.

**Methods:** A time-dependent method was used to estimate *R_t_*, and monthly data from the national TB registry were scrutinized from 2011 to 2021.

**Results:** A decline was observed in SPPTB incidence rates of 50909 SPPTB cases in Iran from 2011 to 2021. Approximately 29.1% of the cases were diagnosed within a month, while 44.5% experienced a one to three-month delay in diagnosis. The analysis revealed substantial heterogeneity in TB transmission dynamics across various provinces of Iran. Provinces such as Sistan and Baluchestan, Golestan, Guilan, Khuzestan, Tehran, and Khorasan Razavi exhibited the highest effective reproduction numbers. Additionally, there was a decreasing trend in the effective reproduction numbers across all provinces from 2011 to 2020.

**Conclusion:** Effective reproduction numbers declined in most provinces from 2011 to 2020 but increased moderately after the COVID-19 pandemic, highlighting the need for targeted public health interventions. Although SPPTB incidence rates are declining nationally, elevated incidence rates and effective reproduction numbers in regions such as Sistan and Baluchestan, Golestan, Guilan, Khuzestan, Tehran, and Khorasan Razavi signify the need for persistent TB management efforts in Iran.

## Background

 Tuberculosis (TB) is a significant global public health challenge with a high incidence worldwide. In 2022, about 10.6 million people were diagnosed with TB, leading to 1.3 million deaths, making it the second leading infectious cause of death after COVID-19. TB predominantly affects adults in their most productive years, with over 85% of cases and deaths occurring in low and middle-income countries.^1-3^ Viral epidemics and pandemics such as severe acute respiratory syndrome (SARS), Middle East respiratory syndrome (MERS), human immunodeficiency virus (HIV), influenza A (H1N1), and COVID-19 predominantly affect the lungs and are transmitted through respiratory droplets.^4^ In contrast, TB remains the top infectious disease killer globally and exhibits an endemic nature in numerous countries, with a more stable transmission pattern.^5^ Although the viral pandemic has received significant global attention and resources, TB has been described as an unseen pandemic, continuing to cause millions of deaths, especially in low and middle-income countries, without receiving commensurate investment and urgency.^6^

 Iran is considered a lower-moderate burden country for TB, with an estimated incidence rate of 13 cases per 100 000 people in 2020.^7^ However, the incidence rate varies widely across different regions of Iran.^8^ The incidence of TB in Iran has decreased in recent years due to various TB control measures implemented in the country. However, Iran continues to face challenges in controlling TB, particularly due to the high incidence of the disease in neighboring countries such as Afghanistan and Pakistan.^9^ The prevalence of TB is higher in the border areas of Iran such as Sistan and Baluchestan and lower in the central regions.^10,11^

 TB is primarily transmitted through the air from person to person. When a person with TB disease of the lungs or throat coughs, speaks, or sings, mycobacterium TB can be released into the air. People nearby may inhale these bacteria and become infected.^12^ Among the different forms of TB, smear-positive pulmonary tuberculosis (SPPTB) stands out as a particularly concerning variant due to its heightened infectiousness. SPPTB has a higher potential for person-to-person transmission compared to other forms of TB, making it a focal point for public health efforts.^13^ Furthermore, patients with SPPTB are at increased risk of spreading the disease to others.^14^

 The effective reproduction number (*R*_t_) is the number of secondary cases generated by an infectious individual in a population that is not completely susceptible to the disease. It considers the current state of the population, including the number of individuals who are already infected or immune to the disease.^15^ The *R*_t_ is a key metric that provides insights into the transmission dynamics of infectious diseases within a population and can guide public health strategies to mitigate the spread of the disease.* R*_t _> 1 indicates that the disease is spreading quickly, necessitating more aggressive measures to reduce transmission such as increased testing, contact tracing, and isolation of infected individuals.^16^ The *R*_t_ for TB varies depending on environmental conditions and the population’s health. A Bayesian melding approach was used to estimate India’s basic *R*_t_ for TB to be 0.92, indicating the slow elimination of TB in India from 2006 to 2011.^17^ Several studies have estimated *R*_t_ for TB in different populations. For example, a study estimated *R*_t_ for TB in the USA to be 0.55 from 1955 to 1994.^18^ Another study estimated a high, *R*_t_, for TBin China to be 4.3.^19^ Furthermore, a Bayesian modeling study estimated *R*_t_ for TB in Iran to be 1.06 ± 0.05 from 2018 to 2022.^20^

 This study aimed to investigate the *R*_t_ of SPPTB in different provinces of Iran between 2011 and 2021 and identify the characteristics of SPPTB patients, including age, gender, bacilli density in the initial smear, delayed diagnosis, and geographical distribution. To date, no studies have reported the *R*_t_ for TB in different provinces of Iran.

## Methods

 This study involved a national historical cohort analysis in Iran, conducted from March 2011 to March 2021, utilizing data from the National Tuberculosis and Leprosy Registration Center of Iran’s Ministry of Health and Medical Education (MOHME). The analysis involved monthly data collection, identifying a total of 106 588 TB cases, of which 50 909 cases were diagnosed with SPPTB during the study period.

###  Study participant 

 The study’s eligibility criteria focused on individuals with SPPTB due to its high transmission rate in the community and prioritization in treatment, resulting in the inclusion of 50 909 SPPTB cases. Demographic characteristics and other pertinent risk factors were extracted based on the information obtained through the TB registration portal. These factors included age, pretreatment weight, gender, delayed diagnosis (time between TB symptom onset and diagnosis), location (urban vs. rural), bacilli density in the initial smear, nationality (Iranian vs. other), and the like. Bacilli density was classified as 1-9 bacilli (determined as 1-9 AFB per 100 immersion fields), 1 + (determined as 10-99 AFB per 100 immersion fields), 2 + (determined as 1-10 AFB per 1 immersion fields), and 3 + (determined as > 10 AFB per 1 immersion fields). The *R*_t_ of SPPTB in Iran and its provinces is illustrated using a GIS map.

###  Statistical analysis

 Quantitative and qualitative variables were summarized as mean ± standard deviation (SD) and frequency (%), respectively. The chi-square goodness-of-fit test was applied to evaluate the uniform distribution of patients across levels of qualitative variables. The data analysis was conducted using statistical software programs, including SPSS (version 22, Institute Inc., Chicago, IL, USA), R (version.4.2.2, www.r-project.org), ArcGIS (version 10), and Excel (version 2019), with a significance level of 0.05. The R0 package in R software was used to estimate the *R*_t_ of SPPTB.^21^

###  Estimating serial interval 

 The World Health Organization (WHO) recommends screening close contacts of people with active TB for TB infection as soon as possible after their exposure to the infected person because the risk of developing TB disease is highest in the first few months after exposure.^22,23^ Close contacts should be screened for TB infection using a tuberculin skin test (TST) or an interferon-gamma release assay (IGRA) within 2-8 weeks after their last exposure to the infectious TB case. If the initial screening test is negative, a repeat test may be recommended 8-10 weeks after exposure.^23^ In Iran, health centers have been gathering data on the TB incidence among individuals in close contact with infected people since 2018. This data analysis is valuable for estimating the serial interval (SI) of SPPTB and determining the disease’s *R*_t_.

 TB has no early symptoms, and an infected person can easily transmit the disease. The incubation period (no symptoms) for TB can vary widely, ranging from a few weeks to several months. On average, symptoms develop approximately 2 to 12 weeks after infection with TB bacteria. However, symptoms may not appear until many months or even years later in some cases.^24^

 Generation time and SI are key epidemiological measures providing insights into the spread of infectious diseases. Generation time is the average duration from symptom onset in an infected individual to symptom onset in those they infect, offering a comprehensive view of transmission dynamics. Accurate estimation of the generation time is crucial for effective disease modeling and public health planning. Researchers often determine the generation time through the SI, which specifically measures the time between symptom onset in a primary case and its secondary case.^25^ Estimating the SI for TB disease can be challenging, particularly if the exact time of symptom onset in the second case is unknown. Methods for estimating the SI include the median serial interval method, the distribution of serial intervals method, and mathematical models.^26^ These methods require high-quality and complete data to provide accurate estimates. The maximum likelihood method has been used in studies to estimate the mean SI for TB, but results should be interpreted cautiously, considering the limitations and uncertainties of the data and methods.^27^

 The average delay between symptom onset and treatment initiation was 10.8 ± 10.7 weeks, while the average delay between disease diagnosis and treatment initiation was about one week. For TB, the end of the infectious period and the incubation period of the disease were considered to occur two weeks after treatment initiation, with an incubation duration of 2-10 weeks. Based on data from 483 primary cases of SPPTB with corresponding secondary cases recorded, our preliminary evaluation of close contact data yielded a mean ± SD estimated SI of approximately 27.5 ± 11.73 weeks. A Gamma distribution was fitted to SI sample data with a shape parameter α = 5.5 and a scale parameter β = 5. We investigated a range of SI values based on previous studies, which should cover a wide spectrum of possible values. A systematic review by Ma et al discovered that the SI for TB ranges from 0.4 years to 1.65 years,^28^ Hence, an external validity analysis of the SI distribution for TB data was conducted. Due to the lack of sufficient data on the onset of symptoms in primary and secondary cases across provinces, a uniform serial interval was assumed for all regions.

###  The effective reproduction number

 The time-dependent method proposed by Wallinga and Teunis was used to estimate *R*_t_ during an infectious disease outbreak. This method analyzes the SI distribution by combining data on the number of cases and the duration of the outbreak, providing an estimate of* R*_t_. This method allows for greater flexibility in estimating *R*_t_.^29,30^ To calculate the probability that a case with onset at a certain time was infected by another case, *p*_ij_ that case *i* with onset at time *ti* was infected by case *j* with onset at time *t*_j_ is calculated as:


$$p_{ij}=\frac{N_{i}w\left(t_{i}-t_{j}\right)}{\sum_{i\neq k}N_{i}w\left(t_{i}-t_{j}\right)}$$

where N is the total number of cases, and *w* is a weight function that decreases with increasing time between the onsets of the cases. *R*_t_ for case *j* is then calculated as:


$$R_{j}=\sum_{j}p_{ij}$$

The average of *R*_t_ for a particular date of onset is calculated as:


$$R_{t}=\frac{1}{N_{t}}\sum_{\left\{t_{j}=t\right\}}R_{j}$$

where *N*_t_ is the number of cases with the same date of onset. The time-dependent method also allows for corrections to real-time estimations, considering the number of secondary cases that have yet to be observed. Furthermore, the method can account for imported cases during the epidemic. In summary, the time-dependent method uses a weighted equation to calculate the likelihood of infection between cases and then uses this information to calculate the *R*_t_ for each case and each date of onset. The method also allows for corrections in real-time estimation and can account for imported cases.

 The time-dependent model for estimating the reproduction number is chosen to capture the dynamic evolution of disease spread over time and provide a more sophisticated understanding compared to static models. This modeling approach accommodates temporal variations, offering a comprehensive insight into the nuanced and evolving disease transmission patterns. Moreover, it allows for the estimation of time-varying reproduction numbers, which provide a real-time picture of disease spread and can help evaluate the impact of intervention strategies.^31^ The rationale for employing the time-dependent model is rooted in the belief that it will yield a more precise estimation of the reproduction number within the specific context of the study. The use of this model will be thoroughly justified in the methods section, ensuring transparency and elucidating the rationale guiding the analytical approach.

 Furthermore, the time-dependent model is particularly relevant for monitoring the spread of infectious diseases such as COVID-19. This approach is also useful for predicting turning points in disease outbreaks and can be applied to noisy surveillance data to estimate local time-varying reproduction numbers.^32,33^ Additionally, using time-dependent models improved the accuracy of estimating the effective reproduction number, which is a key indicator of disease spread.^34^

## Results

Table 1 presents the baseline characteristics of 50 909 patients with SPPTB in Iran and the results of a single risk factor analysis. The mean age of the patients was 51.6 ± 21.3 years, with the highest proportion of patients being in the age group of > 64 years (35.3%). Furthermore, the number of male patients (54.6%) was higher than that of female patients (45.4%), and the average weight of SPPTB patients was 55.1 ± 14.5 kg. Bacilli density in the initial smear was observed in varying proportions, with 3 + (37.0%) being the most common. Moreover, delayed diagnosis was found in 29.1% of patients with SPPTB diagnosed within one month, while 44.5% of patients were diagnosed after one to three months. All variables showed statistical significance differences (*P* < 0.05), and most patients were Iranian (84.3%) and resided in urban areas (67.0%), as depicted in Table 1.

**Table 1:** Baseline characteristics and single risk factor analysis of SPPTB cases in Iran (N = 50909)

**Characteristics**	**SPPTB Patients**		
	**Number**	**Percent**	***P*** ** Value**
**Age (y)**			0.001
**<15**	894	1.8	
**15-35**	14261	28.0	
**36-63**	17779	34.9	
**>64**	17975	35.3	
**Gender**			0.001
**Female**	23108	45.4	
**Male**	27801	54.6	
**Bacilli density in initial smear**			0.001
**1–9 Bacilli**	3727	7.3	
**1+**	17114	33.6	
**2+**	11242	22.1	
**3+**	18826	37.0	
**Delayed diagnosis (month)**			0.001
**<1**	14800	29.1	
**1-3**	22633	44.5	
**>3**	13476	26.5	
**Nationality**			0.001
**Iranian**	42935	84.3	
**Others**	7974	15.7	
**Location**			0.001
**Urban**	34114	67.0	
**Rural**	16795	33.0	

*Note.* SPPTB: Smear-positive pulmonary tuberculosis.

**Figure 1:**
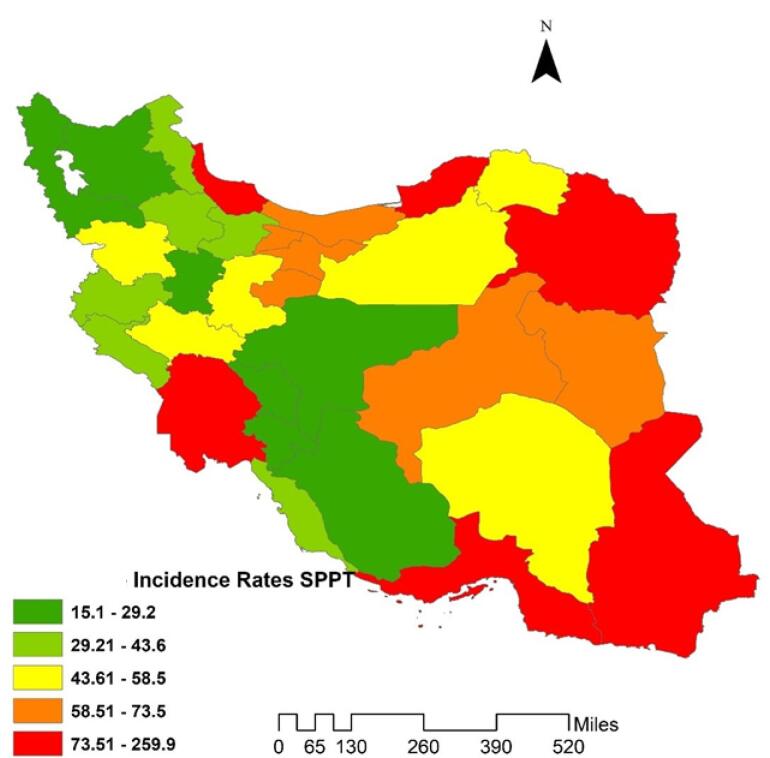
GIS maps of incidence rates of SPPTB in Iranian provinces during 2011-2021. *Note.* SPPTB: Smear-positive pulmonary tuberculosis

 The GIS map presented in this study displays the spatial distribution of SPPTB in different provinces of Iran. The map shows a wide variation in 11-year incidence rates across the country, with some provinces exhibiting high rates while others have relatively low rates. The highest SPPTB incidence rates were observed in Sistan and Baluchestan province, with an incidence rate of 259.9 per 100 000 populations, followed by Golestan (216.6), Khuzestan (96.9), Khorasan Razavi (101.7), and Hormozgan province (89). In contrast, some provinces had lower SPPTB incidence rates such as Chaharmahal and Bakhtiari (15.1), Kohgiluyeh and Boyer-Ahmad (15.5), and Fars (21.7). The overall average SPPTB incidence rate for the country was 68.6 per 100 000 population (Figure 1).

Table 2 presents the effective *R*_t_ of SPPTB in various provinces of Iran from 2011 to 2021. The table consists of 33 Iranian provinces and their respective *R*_t_ values for each year from 2011 to 2021. The *R*_t_ values ranged from a minimum of 0.03 in Kohgiluyeh and Boyer-Ahmad to a maximum of 1.19 in Sistan and Baluchestan, indicating substantial heterogeneity in TB transmission dynamics. Provinces such as Sistan and Baluchestan and Golestan exhibited the highest *R*_t_ values, suggesting persistent TB transmission and the need for targeted interventions to curb disease spread. Conversely, provinces such as Kohgiluyeh and Boyer-Ahmad, Chaharmahal and Bakhtiari, and Ilam demonstrated the lowest *R*_t_ values, indicating relatively lower transmission rates or effective control measures. Additionally, the analysis revealed a decreasing trend in *R*_t_ values from 2011 to 2021 in all provinces.

**Table 2:** Effective reproduction number of SPPTB in Iranian provinces during 2011-2021

**Province **	**2011**	**2012**	**2013**	**2014**	**2015**	**2016**	**2017**	**2018**	**2019**	**2020**	**2021**	**Mean**
**Alborz**	1.06	1.02	1	0.99	1.01	0.96	1.03	1.02	0.8	0.68	1.01	0.96
**Ardabil**	1.07	1.03	1.02	1.01	0.96	0.87	0.76	0.69	0.82	0.66	0.94	0.89
**Bushehr**	1.02	1	0.97	0.91	0.88	0.78	0.69	0.56	0.53	0.67	0.85	0.81
**Chaharmahal and Bakhtiari**	1.01	0.82	0.73	0.68	0.51	0.48	0.45	0.24	0.1	0.12	0.31	0.49
**East Azarbaijan**	1.11	1.01	0.99	0.98	1	0.96	1.01	1	0.85	0.96	0.67	0.96
**Esfahan**	1.09	0.98	1.01	0.98	1.02	1	1.11	1.14	0.84	0.71	0.88	0.98
**Fars**	1.04	1.02	1.01	0.94	1.02	1.01	1.08	1	0.76	0.49	0.78	0.92
**Golestan**	1.15	1.1	1.09	1.07	1.05	1.04	1.03	1.02	1.04	1.03	1.05	1.06
**Guilan**	1.06	1.02	1	1	1.01	1	1.01	0.99	1.01	0.99	1.02	1.01
**Hamedan**	1.05	0,91	0.86	0.72	0.65	0.65	0.61	0.45	0.56	0.28	0.55	0.64
**Hormozgan**	1.04	0.98	1	1.05	0.98	0.99	0.87	0.83	0.79	0.66	1	0.93
**Ilam**	1.01	0.88	0.71	0.62	0.59	0.45	0.41	0.25	0.16	0.18	0.14	0.49
**Kerman**	1.04	0.98	1.02	1	0.99	1	1.06	1.09	0.86	0.92	0.87	0.99
**Kermanshah**	1.01	1	0.97	1.02	1	1.02	0.98	0.81	0.57	0.54	1	0.90
**Khuzestan**	1.04	1.02	1	0.98	1	1.01	1	0.98	1.01	0.96	1.01	1.00
**Kohgiluyeh and Boyer-Ahmad**	1.01	1	0.81	0.64	0.46	0.29	0.2	0.03	0.08	0.08	0.29	0.44
**Kurdistan**	1.12	1.07	0.97	1	1.01	1.05	1.05	0.94	0.71	0.57	0.56	0.91
**Lorestan**	1.06	1.05	0.96	0.97	1.06	0.97	0.96	0.8	0.55	0.52	0.72	0.87
**Markazi**	1.05	1.02	0.99	0.97	1.07	0.96	0.91	0.61	0.46	0.41	0.65	0.83
**Mazandaran**	1.01	0.99	0.96	0.97	0.95	0.84	0.76	0.69	0.88	0.88	1.01	0.90
**North Khorasan**	1.02	0.99	0.97	0.72	0.68	0.65	0.63	0.50	0.74	0.46	0.47	0.71
**Qazvin**	1.03	1.01	1	0.91	0.98	1	0.98	0.54	0.54	0.27	0.37	0.78
**Qom**	1.02	1.04	0.96	1.00	0.98	1.03	0.96	0.65	0.46	0.27	0.50	0.80
**Razavi Khorasan**	1.05	1.03	1	1.01	1	1	1.01	1	1.02	1	1.01	1.01
**Semnan**	1.03	1	0.97	1.02	0.99	0.98	0.91	0.45	0.24	0.21	0.39	0.74
**Sistan and Baluchestan**	1.19	1.16	1.11	1.1	1.08	1.07	1.06	1.05	1.06	1.05	1.07	1.09
**South Khorasan**	1.04	1.02	1.01	0.98	1.01	0.96	1.02	0.67	0.46	0.32	0.49	0.82
**Tehran**	1.05	1.02	1	1.01	1	0.99	0.98	0.97	1	0.98	1.02	1.00
**West Azerbaijan**	1.07	1.05	0.96	0.99	0.99	1	0.98	0.91	0.83	0.67	0.94	0.94
**Yazd**	1.05	1	1	1.01	1	1	1.04	0.78	0.78	0.59	0.87	0.92
**Zanjan**	1.02	1.01	1	0.99	0.96	0.95	0.88	0.52	0.53863	0.2692	0.36	0.77

*Note.* SPPTB: Smear-positive pulmonary tuberculosis.

**Figure 2:**
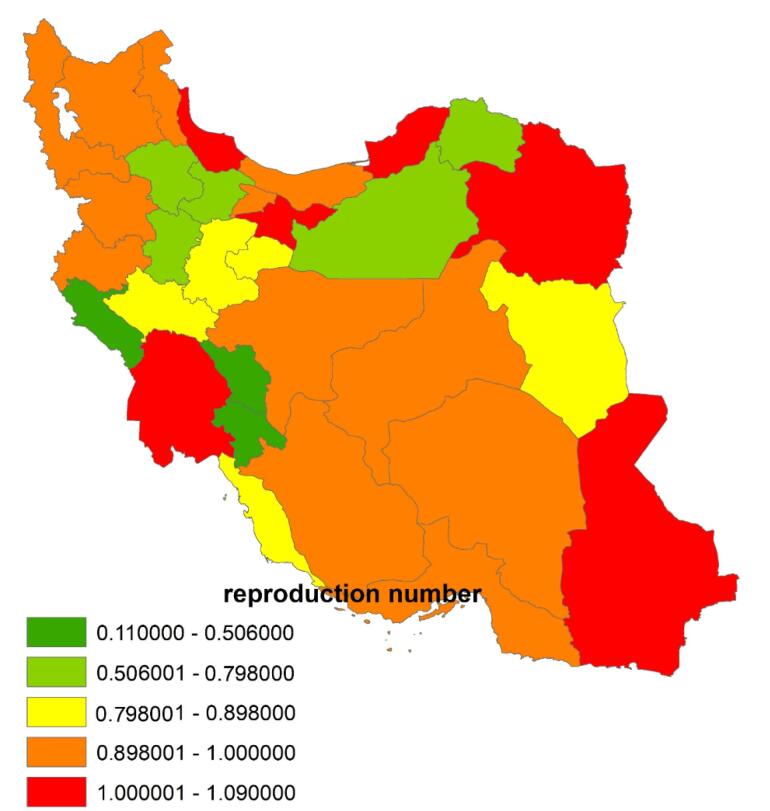
GIS maps of the effective reproduction number of SPPTB in Iranian Provinces during 2011-2021. *Note.* SPPTB: Smear-positive pulmonary tuberculosis

 The spatial distribution of *R*_t_ varied across Iranian provinces. The highest *R*_t_ was observed in Sistan and Baluchestan (1.09), followed by Golestan (1.06), and the lowest in Kohgiluyeh and Boyer-Ahmad (0.44). The mean *R*_t_ value ranged from 0.44 to 1.09. Notably, Sistan and Baluchestan, along with five other provinces, exhibited *R*_t_ values exceeding 1 (Figure 2). This indicates a concerning trend, as an *R*_t_ greater than 1 suggests that TB transmission remains active and may lead to an increase in new TB cases in the future.

**Figure 3:**
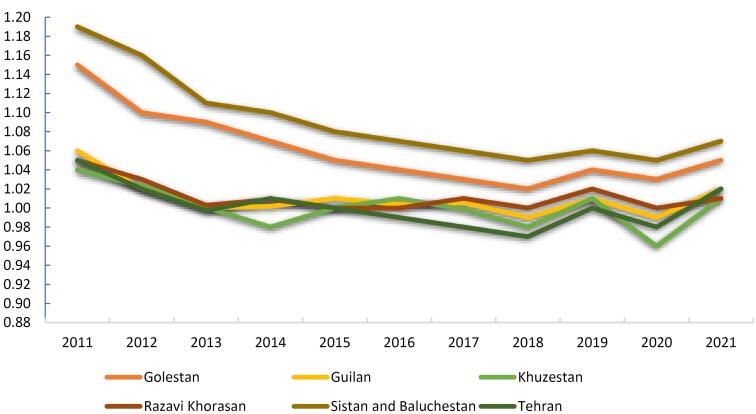
Provinces with average effective reproduction number of SPPTB exceeding 1 during 2011-2021. *Note.* SPPTB: Smear-positive pulmonary tuberculosis

Figure 3 illustrates the provinces where the average *R*_t_ of SPPTB exceeded 1 from 2011 to 2021. Among these provinces, Sistan and Baluchestan had the highest average *R*_t_ of 1.09, indicating sustained transmission of SPPTB, followed by Golestan exhibiting average *R*_t_ values of 1.06, suggesting ongoing transmission dynamics. Additionally, Razavi Khorasan and Guilan had average *R*_t_ values of 1.01, while Khuzestan and Tehran recorded an average *R*_t_ of 1.00. Despite variations in the exact values, all these provinces showed consistent transmission potential with an average *R*_t_ exceeding the threshold of 1, signifying sustained SPPTB transmission within these regions over the studied period.

**Figure 4:**
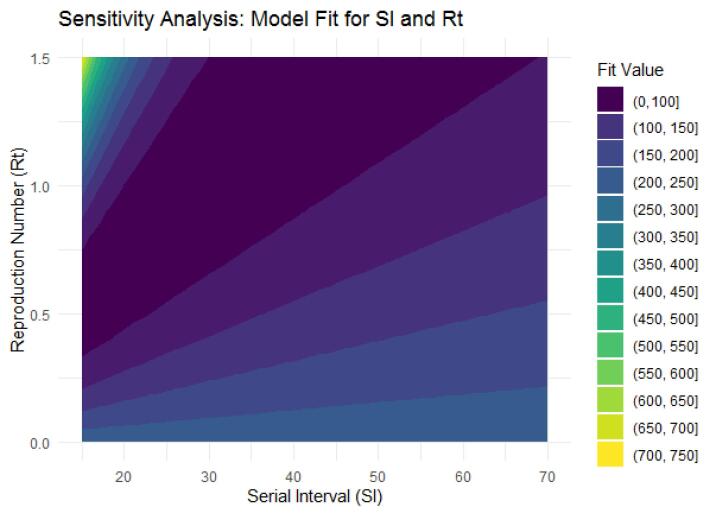
External validity of serial interval (week) and effective reproduction number

 Due to the uncertainty concerning SI, an external validity analysis was conducted by exploring a range of SI values based on prior research, covering a wide spectrum. An external validity analysis encompassed SI values ranging from 15 to 86 weeks and *R*_t_ values from 0 to 2, yielding the best-fit value range [0,100] (dark area), indicating a strong model-data match. Our finding, with an SI of 27.5 weeks and *R*_t_ values between 0.44 to 1.09, fall within this range, thereby corroborating our results (Figure 4).

## Discussion

 The findings of this study indicate that most SPPTB patients in Iran are over 64 years old, with a higher prevalence in male than in female patients. These results are consistent with other studies that have identified age and gender as significant risk factors for TB.^35^ The findings show that the majority of patients with SPPTB are males, which is consistent with previous studies highlighting gender differences in TB incidence rates. The higher incidence of TB among males may be related to behavioral factors and occupational exposure. The mean age of the patients in this study was 51.6 years, with most patients falling into the 36-63 and over 64 age groups, which is consistent with previous studies that reported a higher incidence of TB among older individuals.^36^ The higher incidence of TB among older individuals may be attributed to age-related declines in immune function, increasing their susceptibility to infections.^37^ Moreover, the elderly population is at risk of reactivating latent TB infections acquired in childhood or youth due to age-related immune system decline, which is a critical factor in developing active TB disease in this population and is more important than susceptibility to the initial infection.^38^ Compared to other studies, a study conducted in Iran by Shirazinia et al also reported a high incidence rate of TB in the southeastern regions of Iran, with a higher prevalence of TB in urban areas and older age groups. These findings are consistent with the current study, highlighting the importance of targeting high-risk groups with effective TB control interventions.^39^

 Most patients in this study were Iranian, but a considerable proportion (15.7%) of individuals diagnosed with SPPTB in Iran were non-Iranians, most of whom were of Afghan descent (97%). Sistan and Baluchestan province is home to a large population of Afghan refugees and migrants who may have limited access to TB diagnosis and treatment services.^40^ This province shares borders with Pakistan and Afghanistan and has a unique demographic composition with a significant population of Afghan refugees and migrants. Due to its location and demographic characteristics, the province has been identified as a high-risk area for TB transmission.^41^

 In terms of TB transmission, provinces such as Sistan and Baluchestan, Golestan, Guilan, Khuzestan, Tehran, and Khorasan Razavi generally demonstrate *R*_t_ values close to or slightly above 1, suggesting sustained TB transmission within these regions. Conversely, provinces such as Chaharmahal and Bakhtiari, Ilam and Kohgiluyeh, and Boyer-Ahmad tended to have lower *R*_t_ values, indicating relatively lower levels of TB transmission. Sistan and Baluchestan and Golestan provinces in Iran exhibit elevated TB incidence and mortality rates compared to other regions. This aligns with previous research, which ranked Golestan province as the second highest in terms of TB prevalence in Iran, reporting an incidence rate of 35.9 per 100 000 individuals. Another study also identified Sistan and Baluchestan and Golestan provinces as having the highest TB incidence and mortality rates in Iran, underscoring the pressing need for targeted interventions and improved healthcare infrastructure in these areas to combat TB.^7,42^

 In this study, TB transmission is more pronounced in border regions such as Sistan and Baluchestan, Golestan, Guilan, Khuzestan, and Khorasan Razavi due to a variety of factors, including population dynamics, the state of the healthcare infrastructure, and the effectiveness of TB control measures. These areas may exhibit higher TB prevalence rates due to factors such as heightened mobility, cross-border migration, and vulnerable populations with limited access to healthcare services.^43-45^ In addition, border provinces often experience higher levels of population movement and cross-border migration, which can facilitate the spread of TB. Increased population mobility increases the likelihood of transmission, especially if migrating individuals carry the infection.

 The results provide evidence of the association between migration and TB transmission in various contexts. In South Africa, a study found that migration-adjusted TB prevalence was associated with drug-susceptible TB and rifampicin-resistant TB incidence two years later. Municipalities receiving many migrants with TB had higher future incidence than municipalities receiving fewer migrants. This suggests that cross-municipality migration patterns play a significant role in TB transmission.^46^ In the USA, a case study highlighted the challenges and issues raised by TB in a low-TB-burden setting involving migrants. The case was a 24-year-old Guatemalan immigrant diagnosed with TB after residing in the country for 10 months. The study highlighted the importance of addressing TB in migrant populations, especially in low-TB-burden settings.^47^ In China, internal migration has been identified as a significant factor for TB transmission. The number of migrant TB cases has increased by 30.55% since 2006, with most cases observed in Dalian and Shenyang. Poor traffic conditions, uneven allocation of public health resources, and limited TB knowledge are possible reasons for the high rates of TB in Qinghai and Tibet. Significant positive spatial autocorrelations were found in both the proportion of internal emigrants and immigrants, indicating that people in certain areas are more likely to emigrate to other provinces or that these areas are less attractive for internal immigrants.^48^ A study conducted in 2021 revealed that the spatial cluster incidence map of TB analysis identified Khuzestan Province in western Iran as having substantially higher rates of both total TB and SPPTB compared to neighboring provinces, thus corroborating our findings.^8^ In sum, border provinces with high population movement and cross-border migration are at a higher risk of TB transmission. Effective TB control strategies in these areas should consider the characteristics of internal migration flow and address the challenges faced by migrants in accessing medical care, insurance, and social security.

 Tehran is the capital of Iran and the most populous city in the country. Many individuals migrate to this city for employment opportunities, education, and access to amenities. Similarly, Mashhad, the capital of Khorasan Razavi province, is known as a migrant-receiving city and a religious center with a large number of pilgrims. These factors have contributed to the increased incidence of TB in these two provinces.^8,49,50^ Studies examining TB incidence in China from 2002 to 2018 through an ecological lens revealed a correlation between various environmental factors and the prevalence of TB. These factors include residents’ income, unemployment rates, education levels, availability of medical resources, population density, duration of sunshine exposure, and dietary habits.^51^ Overall, the dynamic demographic and socio-economic landscape of Tehran and Mashhad, characterized by population mobility, urbanization, and diverse socio-cultural activities, creates environments where TB transmission can occur more readily, highlighting the importance of targeted public health interventions and surveillance efforts in these areas.

 Between 2011 and 2020, effective *R*_t_ declined in most provinces but experienced a moderate increase after 2020, coinciding with the onset of the COVID-19 pandemic. The year 2020 will likely be remembered as the year defined by COVID-19 or coronavirus disease. The SARS-CoV-2, which triggered this pandemic, emerged in late 2019 in China. The COVID-19 pandemic had a significant impact on the TB care system, leading to reductions in TB testing and reporting^52^ due to disruptions in TB services and restrictions on patient movement, which resulted in increased TB-related deaths. The COVID-19 outbreak caused a rise in TB cases and fatalities worldwide, affecting the lives and livelihoods of millions of people. Consequently, the COVID-19 pandemic reversed a decade of progress in TB epidemic control.^20^

 The study relied on data from a national TB registry, which might have limitations in terms of completeness and accuracy. Additionally, the availability of certain data points such as the time of symptom onset for primary and secondary cases in every province was limited, potentially impacting the accuracy of the analysis. Variations in SI values might exist across different regions or populations, and a broader dataset could provide more nuanced insights into SI distribution and its impact on the model. The study utilized a specific modeling approach to estimate *R*_t_ and analyze TB transmission dynamics. While commonly used, this approach relies on certain assumptions about TB transmission patterns and may not fully capture the complexity of transmission dynamics in all settings. The generalizability of the findings of the study beyond the context of Iran may be limited as factors influencing TB transmission dynamics (e.g., healthcare infrastructure, population demographics, and TB control measures) may vary in different countries or regions, affecting the applicability of the study’s findings elsewhere.

**Highlights**

- Effective reproduction numbers declined in most provinces from 2011 to 2020, but it showed a moderate increase after the COVID-19 pandemic
- TB prevalence is higher among older males, with delayed diagnosis being common.
- The highest rates of SPPTB transmission were observed in six Iranian provinces: Sistan and Baluchestan, Golestan, Guilan, Khuzestan, Tehran, and Khorasan Razavi.
- Persistent TB control efforts are needed in high-incidence regions.

## Conclusion

 Effective *R*_t_ declined in most provinces from 2011 to 2020, but it exhibited a moderate increase after the COVID-19 pandemic. This study also found that SPPTB in Iran is more prevalent among older males, with delayed diagnosis being common. A significant proportion of non-Iranians, mostly of Afghan descent, were diagnosed with SPPTB. This study underscores the importance of understanding the transmission dynamics of TB to develop targeted public health interventions in regions by calculating *R*_t_ values. The mean *R*_t_ for most provinces was fluctuating around 1, indicating the endemic nature of TB in the country and suggesting that efforts to disrupt TB transmission have yet to yield the desired results, raising concerns about a potential increase in future TB cases. This underscores the importance of monitoring and addressing the underlying factors contributing to elevated *R*_t_, especially in provinces with higher TB transmission rates. Provinces such as Sistan and Baluchestan, located near Pakistan and Afghanistan, experience a higher transmission of TB disease. Additionally, border provinces and densely populated regions like Tehran and Khorasan Razavi exhibit a higher propensity for TB transmission than other provinces. Moreover, temporal trends can be observed within provinces, with fluctuations in *R*_t_values occurring over the years due to various factors such as changes in healthcare infrastructure, population dynamics, and the implementation of TB control measures. This information provides insights for public health strategies tailored to specific regions, considering their unique epidemiological challenges.

## Acknowledgments

 The authors express their gratitude and appreciation to the Tuberculosis and Leprosy Registration Center of Iran’s Ministry of Health and Medical Education for providing data. Moreover, they would like to thank the Vice-Chancellor for Research and Technology at Mashhad University of Medical Sciences (MUMS).

## Authors’ Contribution

**Conceptualization:** Maryam Rastegar.

**Data curation:** Eisa Nazar, Mahshid Nasehi, Saeed Sharafi.

**Formal analysis:** Maryam Rastegar.

**Funding acquisition:** Mohammad Taghi Shakeri.

**Investigation:** Vahid Fakoor.

**Methodology:** Vahid Fakoor, Maryam Rastegar.

**Project administration:** Mohammad Taghi Shakeri.

**Resources:** Maryam Rastegar.

**Software:** Maryam Rastegar.

**Supervision:** Mohammad Taghi Shakeri, Vahid Fakoor.

**Validation:** Mohammad Taghi Shakeri, Vahid Fakoor.

**Visualization:** Maryam Rastegar.

**Writing–original draft:** Maryam Rastegar.

**Writing–review & editing:** Mohammad Taghi Shakeri, Vahid Fakoor, Eisa Nazar.

## Competing Interests

 The authors declare that they have no conflict of interests.

## Ethical Approval

 The study received approval from the Research Ethics Committee of Mashhad University of Medical Sciences (Code: IR.MUMS.FHMPM.REC.1401.052). All methods were conducted according to relevant guidelines and regulations. Informed consent was waived by the Research Ethics Committee of Mashhad University of Medical Sciences.

## Funding

 This study was financially supported by the Vice-Chancellor for Research and Technology, Mashhad University of Medical Sciences, Iran (Project No. 4001759).

## References

[1] Bagcchi S (2023). WHO’s global tuberculosis report 2022. Lancet Microbe.

[2] Portnoy A, Arcand JL, Clark RA, Weerasuriya CK, Mukandavire C, Bakker R (2023). The potential impact of novel tuberculosis vaccine introduction on economic growth in low- and middle-income countries: a modeling study. PLoS Med.

[3] Seung KJ, Keshavjee S, Rich ML (2015). Multidrug-resistant tuberculosis and extensively drug-resistant tuberculosis. Cold Spring Harb Perspect Med.

[4] Ong CWM, Migliori GB, Raviglione M, MacGregor-Skinner G, Sotgiu G, Alffenaar JW (2020). Epidemic and pandemic viral infections: impact on tuberculosis and the lung: a consensus by the World Association for Infectious Diseases and Immunological Disorders (WAidid), Global Tuberculosis Network (GTN), and members of the European Society of Clinical Microbiology and Infectious Diseases Study Group for Mycobacterial Infections (ESGMYC). Eur Respir J.

[5] Gopalaswamy R, Shanmugam S, Mondal R, Subbian S (2020). Of tuberculosis and non-tuberculous mycobacterial infections - a comparative analysis of epidemiology, diagnosis and treatment. J Biomed Sci.

[6] Mehra C. The twin epidemics: TB and COVID-19 in India. In: Health Dimensions of COVID-19 in India and Beyond. Singapore: Springer Nature Singapore; 2022. p. 83-97.

[7] Fallahzadeh H, Khazaei Z, Najafi ML, Rahimi Pordanjani S, Goodarzi E (2023). Distribution incidence, mortality of tuberculosis and human development index in Iran: estimates from the global burden of disease study 2019. BMC Public Health.

[8] Kiani B, Raouf Rahmati A, Bergquist R, Hashtarkhani S, Firouraghi N, Bagheri N (2021). Spatio-temporal epidemiology of the tuberculosis incidence rate in Iran 2008 to 2018. BMC Public Health.

[9] Tavakoli A (2017). Incidence and prevalence of tuberculosis in Iran and neighboring countries. Zahedan J Res Med Sci.

[10] Ghaffari-Fam S, Hosseini SR, Heydari H, Vaseghi-Amiri R, Daemi A, Nikbakht HA (2015). Epidemiological patterns of tuberculosis disease in the Babol, Iran. J Anal Res Clin Med.

[11] Masjedi MR, Farnia P, Sorooch S, Valiollah Pooramiri M, Mansoori SD, Zia Zarifi A (2006). Extensively drug-resistant tuberculosis: 2 years of surveillance in Iran. Clin Infect Dis.

[12] World Health Organization (WHO). WHO Guidelines on Tuberculosis Infection Prevention and Control: 2019 Update. Geneva: WHO; 2019. 30933444

[13] Nardell EA (2015). Transmission and institutional infection control of tuberculosis. Cold Spring Harb Perspect Med.

[14] Ahmad N, Baharom M, Aizuddin AN, Ramli R (2021). Sex-related differences in smear-positive pulmonary tuberculosis patients in Kuala Lumpur, Malaysia: prevalence and associated factors. PLoS One.

[15] Achaiah NC, Subbarajasetty SB, Shetty RM (2020). R(0) and R(e) of COVID-19: can we predict when the pandemic outbreak will be contained?. Indian J Crit Care Med.

[16] Lim JS, Cho SI, Ryu S, Pak SI (2020). Interpretation of the basic and effective reproduction number. J Prev Med Public Health.

[17] Narula P, Azad S, Lio P (2015). Bayesian melding approach to estimate the reproduction number for tuberculosis transmission in Indian states and union territories. Asia Pac J Public Health.

[18] Salpeter EE, Salpeter SR (1998). Mathematical model for the epidemiology of tuberculosis, with estimates of the reproductive number and infection-delay function. Am J Epidemiol.

[19] Ma Y, Jenkins HE, Sebastiani P, Ellner JJ, Jones-López EC, Dietze R (2020). Using cure models to estimate the serial interval of tuberculosis with limited follow-up. Am J Epidemiol.

[20] Rastegar M, Nazar E, Nasehi M, Sharafi S, Fakoor V, Shakeri MT (2024). Bayesian estimation of the time-varying reproduction number for pulmonary tuberculosis in Iran: a registry-based study from 2018 to 2022 using new smear-positive cases. Infect Dis Model.

[21] Obadia T, Haneef R, Boëlle PY (2012). The R0 package: a toolbox to estimate reproduction numbers for epidemic outbreaks. BMC Med Inform Decis Mak.

[22] World Health Organization (WHO). WHO Policy on TB Infection Control in Health-Care Facilities, Congregate Settings and Households. Geneva: WHO; 2009. 24432438

[23] Petersen E, Chakaya J, Jawad FM, Ippolito G, Zumla A (2019). Latent tuberculosis infection: diagnostic tests and when to treat. Lancet Infect Dis.

[24] Borgdorff MW, Sebek M, Geskus RB, Kremer K, Kalisvaart N, van Soolingen D (2011). The incubation period distribution of tuberculosis estimated with a molecular epidemiological approach. Int J Epidemiol.

[25] McAloon CG, Wall P, Griffin J, Casey M, Barber A, Codd M (2021). Estimation of the serial interval and proportion of pre-symptomatic transmission events of COVID- 19 in Ireland using contact tracing data. BMC Public Health.

[26] Nishiura H (2007). Early efforts in modeling the incubation period of infectious diseases with an acute course of illness. Emerg Themes Epidemiol.

[27] Vynnycky E, Fine PE (2000). Lifetime risks, incubation period, and serial interval of tuberculosis. Am J Epidemiol.

[28] Ma Y, Horsburgh CR, White LF, Jenkins HE (2018). Quantifying TB transmission: a systematic review of reproduction number and serial interval estimates for tuberculosis. Epidemiol Infect.

[29] Wallinga J, Teunis P (2004). Different epidemic curves for severe acute respiratory syndrome reveal similar impacts of control measures. Am J Epidemiol.

[30] Wallinga J, Lipsitch M (2007). How generation intervals shape the relationship between growth rates and reproductive numbers. Proc Biol Sci.

[31] Hong HG, Li Y (2020). Estimation of time-varying reproduction numbers underlying epidemiological processes: a new statistical tool for the COVID-19 pandemic. PLoS One.

[32] Li W, Bulekova K, Gregor B, White LF, Kolaczyk ED (2022). Estimation of local time-varying reproduction numbers in noisy surveillance data. Philos Trans A Math Phys Eng Sci.

[33] An Q, Wu J, Bai JJ, Li X (2022). Using time-dependent reproduction number to predict turning points of COVID-19 outbreak in Dalian, Liaoning province, China. BMC Infect Dis.

[34] Setianto S, Hidayat D (2023). Modeling the time-dependent transmission rate using gaussian pulses for analyzing the COVID-19 outbreaks in the world. Sci Rep.

[35] Kirenga BJ, Ssengooba W, Muwonge C, Nakiyingi L, Kyaligonza S, Kasozi S (2015). Tuberculosis risk factors among tuberculosis patients in Kampala, Uganda: implications for tuberculosis control. BMC Public Health.

[36] Meysami A, Salehi M, Sargolzaei N (2010). Trend of smear positive pulmonary tuberculosis in Sistan and Baluchestan Province (2005-2008). Zahedan J Res Med Sci.

[37] Piergallini TJ, Turner J (2018). Tuberculosis in the elderly: why inflammation matters. Exp Gerontol.

[38] Kiazyk S, Ball TB (2017). Latent tuberculosis infection: an overview. Can Commun Dis Rep.

[39] Shirazinia R, Saadati D, Zeinali E, Panahi Mishkar A (2017). The incidence and epidemiology of tuberculosis in Sistan region: an update to past researches. Int J Basic Sci Med.

[40] Proença R, Mattos Souza F, Lisboa Bastos M, Caetano R, Braga JU, Faerstein E (2020). Active and latent tuberculosis in refugees and asylum seekers: a systematic review and meta-analysis. BMC Public Health.

[41] Pourhossein B, Doosti Irani A, Mostafavi E (2015). Major infectious diseases affecting the Afghan immigrant population of Iran: a systematic review and meta-analysis. Epidemiol Health.

[42] Honarvar MR, Charkazi A, Mirkarimi K, Sheikhi M, Kamalinia HR, Rahim Arbabi E (2020). Eleven-year epidemiological study of tuberculosis in Golestan province, northern of Iran. Iran J Public Health.

[43] Zahedi Bialvaei A, Asgharzadeh M, Aghazadeh M, Nourazarian M, Samadi Kafil H (2017). Challenges of tuberculosis in Iran. Jundishapur J Microbiol.

[44] Wikell A, Jonsson J, Dyrdak R, Henningsson AJ, Eringfält A, Kjerstadius T (2021). The impact of borderline QuantiFERON-TB Gold Plus results for latent tuberculosis screening under routine conditions in a low-endemicity setting. J Clin Microbiol.

[45] Ssengooba F, Babirye S, Tuhebwe D, Ssennyonjo A, Ssendagire S, Rutaroh A (2022). The right of access to healthcare: an analysis of how legal and institutional frameworks constrain or facilitate access to healthcare for residents in border areas in the East African Community. Int J Equity Health.

[46] Fofana AM, Moultrie H, Scott L, Jacobson KR, Shapiro AN, Dor G (2023). Cross-municipality migration and spread of tuberculosis in South Africa. Sci Rep.

[47] Garcia D, Wares F, Zuroweste E, Guerin P. Tuberculosis and migration. In: Tuberculosis. Elsevier; 2009. p. 892-900. 10.1016/b978-1-4160-3988-4.00100-1.

[48] Liao WB, Ju K, Gao YM, Pan J (2020). The association between internal migration and pulmonary tuberculosis in China, 2005-2015: a spatial analysis. Infect Dis Poverty.

[49] Yazdani-Charati J, Mahaki B, Ahmadi-Basiri E (2017). Identification of high and low-risk areas of tuberculosis in Lorestan province, west of Iran. Tanaffos.

[50] Nazar E, Baghishani H, Doosti H, Ghavami V, Aryan E, Nasehi M (2020). Bayesian spatial survival analysis of duration to cure among new smear-positive pulmonary tuberculosis (PTB) patients in Iran, during 2011-2018. Int J Environ Res Public Health.

[51] Zhang Q, Song W, Liu S, An Q, Tao N, Zhu X (2021). An ecological study of tuberculosis incidence in China, from 2002 to 2018. Front Public Health.

[52] Cioboata R, Biciusca V, Olteanu M, Vasile CM (2023). COVID-19 and tuberculosis: unveiling the dual threat and shared solutions perspective. J Clin Med.

